# Dissociation between reduced diaphragm inspiratory motion and normal diaphragm thickening in acute chronic pulmonary obstructive disease exacerbation: a case report

**DOI:** 10.1097/MD.0000000000019390

**Published:** 2020-03-06

**Authors:** Julien Kracht, Adam Ogna, Abdallah Fayssoil

**Affiliations:** a Unité de Soins Intensifs Cardiologiques, Centre Hospitalier Centre Bretagne, Pontivy, France; b Servizio di pneumologia, Ospedale La Carità, EOC, Locarno, Switzerland; c Institut de Myologie, CHU Pitié Salpetrière, APHP, Paris, France.

**Keywords:** COPD, diaphragm, ultrasound

## Abstract

**Introduction:**

Patients with chronic pulmonary obstructive disease (COPD) are at risk of acute exacerbation. Diaphragm muscle is classically highly solicited in COPD exacerbation.

**Patient concerns:**

A COPD patient was admitted because of acute dyspnea with wheezing.

**Diagnosis:**

acute COPD exacerbation.

**Interventions:**

A diaphragm ultrasound and a Doppler echocardiography were performed at bedside.

**Outcomes:**

We measured diaphragm thickening at the apposition zone and diaphragm inspiratory motion from the subcostal view, in addition with classical echocardiographic parameters.

**Conclusion:**

Despite a normal diaphragm thickening, diaphragm motion during inspiration is reduced in acute COPD exacerbation. These apparently discrepant findings may be explained by the alterations of the respiratory mechanics during COPD exacerbations, which should be considered when evaluating the diaphragmatic function by imaging.

## 1 Introduction

Patients with chronic obstructive pulmonary disease (COPD) are at risk of acute exacerbations, which are associated with a high burden of morbidity and mortality. COPD exacerbations are characterized by a sudden worsening of the respiratory symptoms, due to an aggravation of the bronchial obstruction. As a consequence, the work of breathing increases and the diaphragm muscle is highly solicited, becoming a key determinant in acute situation. Diaphragm function can be assessed at the bedside using ultrasound.^[1]^ In COPD patients admitted to the hospital because of COPD exacerbations, diaphragm ultrasound has been used to predict the success of noninvasive ventilation (NIV).^[2,3]^ Here, we report the diaphragm ultrasound pattern in a patient with COPD acute exacerbation in order to discuss the clinical implications of the previous studies. The patient has provided informed consent for publication of the case.

## 2 Case report

**Figure 1:**
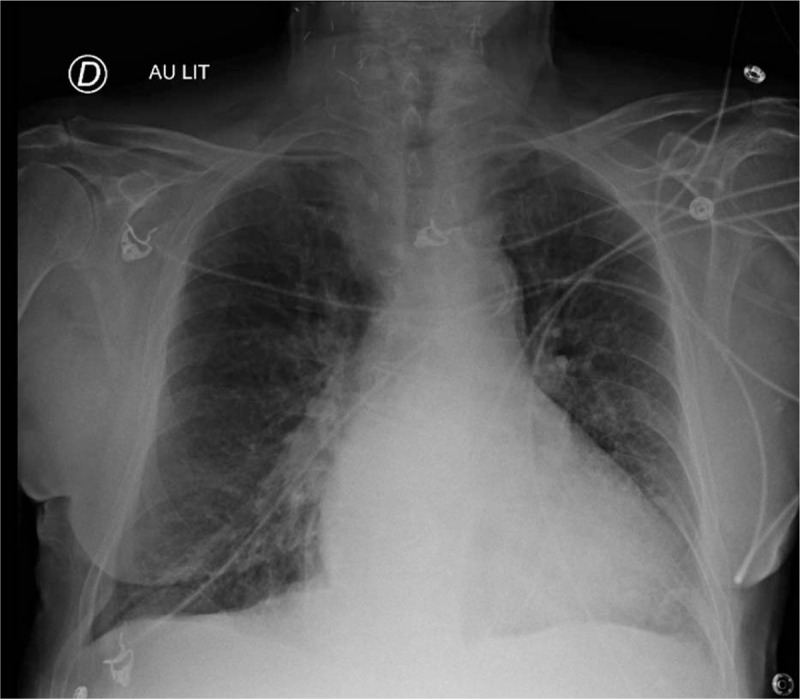
Chest X-ray at admission showing thoracic distension.

A 86 year old patient was admitted to the intensive care unit (ICU) because of acute respiratory insufficiency. His past medical history was significant for COPD, atrial fibrillation, and systemic arterial hypertension. His medication consisted in daily oral anticoagulant, antiplatelet drug, beta-blocker and angiotensin converting enzyme inhibitor. At admission clinical findings were: normal body mass index 25 kg/m^2^, high systemic blood pressure (200/86 mm Hg), tachycardia (110 beat per minute), fever, tachypnea, and severe dyspnea associated with expiratory braking and wheezing. Arterial blood gas analysis disclosed a respiratory acidosis with pH 7.18, and partial pressure of carbon dioxide 67 mm Hg, bicarbonates 25 mmol/L, and lactates 1.9 mmol/L. The other biological results were as follow: brain natriuretic peptid 1267 pg/mL, troponin 14 pg/mL, hemoglobin 12.6 g/dL, white blood cells 18,7 G/L, creatinine 11 mg/L, and C reactive protein 92 mg/L. Electrocardiogram confirmed atrial fibrillation. Chest X-ray disclosed thoracic distension (Fig. 1). Doppler - Echocardiography found normal left ventricular systolic function, subnormal left ventricular diastolic loading (E/Ea at 10), and discretely increased systolic arterial pulmonary pressure (50 mm Hg).

The diagnosis of an acute infectious COPD exacerbation was retained, and the patient was immediately treated with NIV, administration of steroids, inhaled bronchodilators and antibiotics.

**Figure 2:**
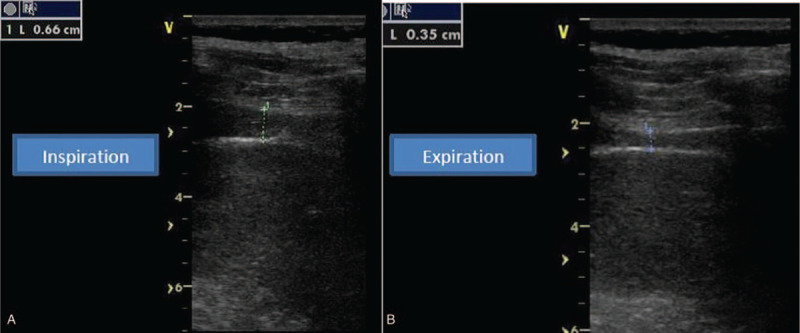
(A and B) Right diaphragm thickness at expiration (left) and at deep inspiration (right).

**Figure 3:**
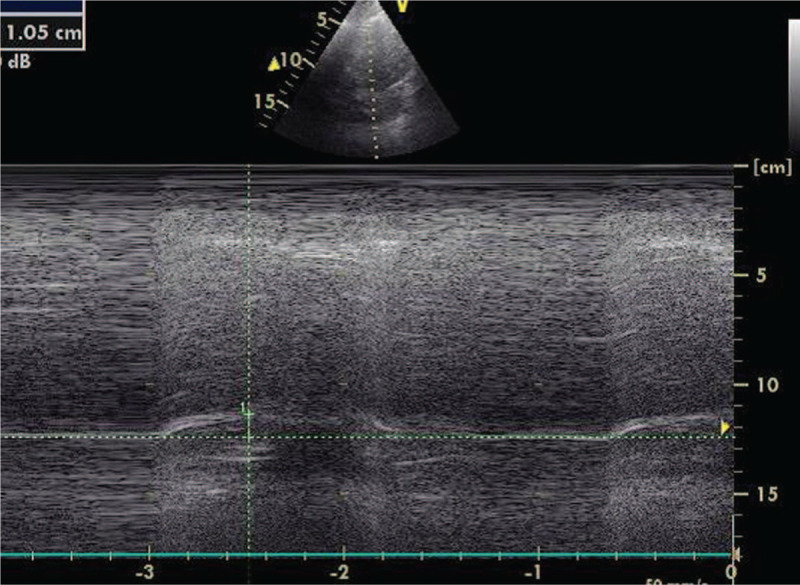
Reduced diaphragm motion at deep inspiration.

Diaphragm ultrasound was performed during noninvasive ventilation. From the subcostal view, with a cardiac probe placed within the mid clavicular line, the diaphragm inspiratory motion was measured using M mode tracing; also, from the mid axillary line closer the costo-phrenic sinus, we measured, using a linear probe, the diaphragm thickness and thickening at the apposition zone (Figs. 2A and B). The diaphragm was identified as a hypo-echogenic muscular layer surrounded by 2 echogenic layer, namely the pleura and the peritoneum.^[4]^ We found a normal diaphragm thickening (ratio between diaphragm thickness at deep inspiration/end expiratory diaphragm thickness of 1.9 for the right diaphragm and 2 for the left diaphragm) whereas the right and left hemi diaphragm motion at deep inspiration were reduced, respectively reaching 15 mm and 10 mm (Fig. 3).

In the follow-up the patient's respiratory situation improved and he could be weaned from NIV and discharged from the hospital.

## 3 Discussion

In the reported case, we found a dissociation between a preserved diaphragm thickening and a pathologically decreased diaphragm inspiratory motion. According to the previous studies, the preserved diaphragm thickening would suggest a preserved diaphragmatic function and should predict a successful NIV weaning and a shorter ICU stay.^[3]^ This is however in contrast with the pathologically decreased diaphragm inspiratory motion, which was reported to be an early predictor of NIV failure.^[2]^

These apparently discrepant findings may be explained by the alterations of the respiratory mechanics during COPD exacerbations, which should be considered when evaluating the diaphragmatic function by imaging.

The action of the normal diaphragm is to provide a piston-like caudal displacement of its dome during the inspiratory contraction phase, increasing the intrathoracic space.^[5]^ Ultrasound imaging allows to noninvasively assess both characteristics of the diaphragm function at the bedside: diaphragm muscular thickening and diaphragm inspiratory motion. Both parameters usually change in a congruent manner in case of diaphragm dysfunction, with a decrease of the inspiratory muscle thickening and a resulting reduction of the caudal displacement of the diaphragm dome.

In COPD patients, the chronic inflammatory airway modifications cause a non-reversible bronchial obstruction, and the frequently associated lung emphysema reduces the elastic recoil forces of the lung. The resulting expiratory flow limitation leads to air trapping and results in a hyperinflation state.^[6]^ In acute COPD exacerbation, the expiratory flow is further limited, and the ventilatory demand is increased. The combination of tachypnea and limited expiratory flow results in a dynamic hyperinflation which worsens the pathological situation. In this condition, the patient displays a tidal breathing closer to total lung capacity and the diaphragm dome is flattened, showing a reduced inspiratory motion due to hyperinflation.^[6,7]^ In the meantime, the inspiratory muscles must provide a higher pressure in order to overcome the increased load of the respiratory system resulting from the abnormal shape of the diaphragm and the reduction of the apposition zone.^[7,8,9,10]^ During ultrasound imaging, the inspiratory diaphragm thickening is thus expected to be preserved. In ICU, the diaphragm thickening reflects the work of breathing in patients on NIV.^[11]^ The acute overload of the diaphragm muscle may lead to diaphragm dysfunction,^[5,12]^ and up to 24% of patients with an acute COPD exacerbation have been reported to develop a diaphragm dysfunction (defined by a diaphragm thickness change during inspiration <20%).^[13]^ Such a sonographically diagnosed diaphragm dysfunction was correlated with NIV failure, longer ICU stay, prolonged mechanical ventilation, need for tracheostomy and increased short term mortality.^[3]^

Histologically, the reduction of the diaphragm muscle strength in COPD may be in relation with sarcomere disruption, caused by the high inspiratory load.^[14]^ Loss of myosin content has been found in COPD diaphragm fibers.^[15]^ Also, the oxidative stress in the diaphragm muscle has been reported to be increased in COPD and this feature was negatively associated with muscle strength.^[16]^ Finally, a production of cytokines been reported in diaphragm fibers of rats in loading situations, which may influence diaphragm function.

According to the above described mechanisms, we interpret the dissociation between the preserved diaphragm thickening and the pathologically decreased diaphragm inspiratory motion we observed in our patient as the manifestation of 2 distinct pathophysiological phenomena with different prognostic value: the preserved inspiratory diaphragmatic thickening suggests a normal muscular function of the diaphragm, whilst the reduced inspiratory motion indicates a hyperinflation status, without giving a valuable evaluation of the muscular function of the diaphragm.

In conclusion, ultrasound imaging may be a useful tool to assess the diaphragm function at the bedside in COPD patients, but the interpretation of the results requires to consider the changes in the respiratory mechanics occurring during an acute COPD exacerbation.

## Author contributions

**Conceptualization:** Abdallah Fayssoil.

**Data curation:** Abdallah Fayssoil.

**Formal analysis:** Abdallah Fayssoil.

**Funding acquisition:** Abdallah Fayssoil.

**Investigation:** Abdallah Fayssoil.

**Methodology:** Abdallah Fayssoil.

**Resources:** Julien Kracht.

**Validation:** Abdallah Fayssoil.

**Writing – original draft:** Abdallah Fayssoil.

**Writing – review and editing:** Abdallah Fayssoil.
